# Ultraviolet laser photolysis of hydrocarbons for nondiamond carbon suppression in chemical vapor deposition of diamond films

**DOI:** 10.1038/lsa.2017.177

**Published:** 2018-04-06

**Authors:** Li-Sha Fan, Loic Constantin, Da-wei Li, Lei Liu, Kamran Keramatnejad, Clio Azina, Xi Huang, Hossein Rabiee Golgir, Yao Lu, Zahra Ahmadi, Fei Wang, Jeffrey Shield, Bai Cui, Jean-Francois Silvain, Yong-Feng Lu

**Affiliations:** 1 Department of Electrical and Computer Engineering, University of Nebraska-Lincoln, Lincoln, NE 68588, USA; 2 Institut de Chimie de la Matière Condensée de Bordeaux—ICMCB-CNRS 87, Pessac, 33608, France; 3 Department of Mechanical and Materials Engineering, University of Nebraska-Lincoln, Lincoln, NE 68588, USA

**Keywords:** combustion chemical vapor deposition, diamond, photolysis, ultraviolet laser

## Abstract

In this work, we demonstrate that ultraviolet (UV) laser photolysis of hydrocarbon species alters the flame chemistry such that it promotes the diamond growth rate and film quality. Optical emission spectroscopy and laser-induced fluorescence demonstrate that direct UV laser irradiation of a diamond-forming combustion flame produces a large amount of reactive species that play critical roles in diamond growth, thereby leading to enhanced diamond growth. The diamond growth rate is more than doubled, and diamond quality is improved by 4.2%. Investigation of the diamond nucleation process suggests that the diamond nucleation time is significantly shortened and nondiamond carbon accumulation is greatly suppressed with UV laser irradiation of the combustion flame in a laser-parallel-to-substrate geometry. A narrow amorphous carbon transition zone, averaging 4 nm in thickness, is identified at the film–substrate interface area using transmission electron microscopy, confirming the suppression effect of UV laser irradiation on nondiamond carbon formation. The discovery of the advantages of UV photochemistry in diamond growth is of great significance for vastly improving the synthesis of a broad range of technically important materials.

## Introduction

**Figure 1:**
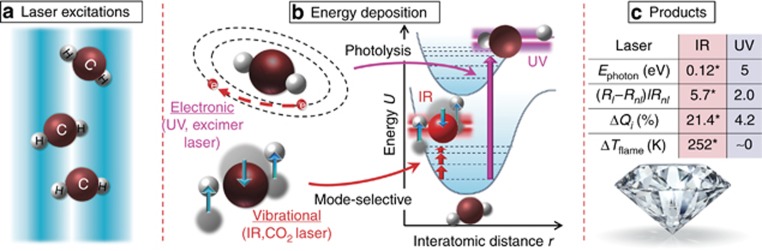
Schematic illustration of how different energy coupling pathways affect diamond synthesis. (**a**) Chemical reactions can be promoted by activating precursor molecules with laser excitations. (**b**) Different energy coupling pathways enabled by laser excitation of specific transitions, IR laser for vibrational excitation, and UV laser for electronic excitations/photolysis. (**c**) Comparison of diamond growth results (*E*_photon_: photon energy, *R*_l_ and *R*_nl_: growth rate with and without laser, Δ*Q*_i_: diamond quality factor increment, and Δ*T*_flame_: flame temperature increment) with IR or UV laser irradiation. *Denotes data from Ref. 7.

The pathway by which energy is transferred to a reactive gas mixture alters the chemical reaction rates^1, 2^. Heating represents the conversion of energy to the translational motion of reactant molecules; that energy is then redistributed among various internal molecular modes, including rotation, vibration and electronic excitation, as illustrated in Figure 1. Because chemical reactions proceed through vibrationally excited or electronically excited states, the conventional heating strategy is low in energy efficiency and process selectivity. With the significant advances in laser technology, lasers provide a unique means of selectively driving chemical reactions by exciting specific transitions in reactant molecules. Extensive experiments have shown that product pathways can be controlled, or even steered, by irradiating one or more laser beams into a reactive gas mixture^3, 4, 5^.

Lasers efficiently stimulate internal modes of gas molecules to sustain a highly nonequilibrium environment, leading to some control over selectivity. As illustrated in Figure 1, electronic excitation or direct photolysis of simple molecules occurs by absorbing photons in the ultraviolet (UV) region, in which the primary products are generally either electronically excited molecules or their dissociation products, that is, reactive species^6^. Vibrational excitation of reactant molecules can be achieved by the absorption of infrared (IR) photons because the molecular vibrational modes are located in the IR region (Figure 1). Using diamond as a sample system, we have demonstrated the significant role that vibrational excitation of precursor molecules plays in improving diamond growth by irradiating a diamond-forming gas phase with a high-power IR laser^7, 8, 9, 10^.

In this context, an intriguing question arises, ‘What happens to diamond growth if electronic excitations are triggered with UV laser irradiation in the gas phase?’ UV photochemistry has long been exploited as a means of gaining chemical control in molecular reactions motivated by suppressing side product channels to obtain the desired deposit. However, there have been few successes in practical material synthesis because the photochemical effects have been believed to be too small^11, 12, 13^. However, selectivity among various competing chemical processes in material synthesis is attractive because it enables a better understanding of the reacting channels, leading to process control and improvements.

As one of the most widely used synthesis techniques, chemical vapor deposition (CVD) relies mostly on phenomena near thermal equilibrium to activate precursor gases and induce gas reactions^11^. A comparative study of the influence of IR laser and UV laser photochemistry on the diamond CVD process provides an example of the effects of these different energy coupling paths in material synthesis. Several attempts have been made to use UV photolysis in diamond synthesis^14, 15, 16, 17, 18^. Kitahama *et al*^17^ reported using a 193-nm laser to photodissociate the acetylene precursor for diamond synthesis. However, subsequent characterization showed the deposition to be amorphous carbon, and the initial work was retracted^15^. Govo *et al*^15^ reported successful diamond growth by irradiating carbon tetrachloride gas using a 193-nm laser, but the process was assisted by predissociated hydrogen. These studies suggest that although UV laser irradiation effectively dissociated the precursor molecules, UV-laser-induced photolysis alone failed to provide a suitable chemical and transport environment for diamond formation.

In this work, instead of simply relying on UV laser photolysis, we introduced UV laser irradiation into a conventional combustion diamond CVD process, demonstrating that the flame chemistry was altered such that it favored diamond growth and suppressed nondiamond carbon accumulation. Optical emission spectroscopy (OES) and laser-induced fluorescence (LIF) demonstrated that UV laser irradiation of the combustion flame promoted the generation of reactive species that are critical to diamond growth, leading to enhanced diamond growth. The growth rate and the film quality were significantly enhanced. Cross-sections of the microstructures of the diamond films revealed fast lateral grain size evolution rates as well as significantly shortened nucleation times with UV laser irradiation, suggesting that secondary nucleation, which is closely related to the accumulation of amorphous graphitic carbon, was significantly suppressed. A narrow amorphous carbon transition zone, averaging 4 nm in thickness, was identified at the film–substrate interface using transmission electron microscopy (TEM), confirming the suppression effect of UV laser irradiation on nondiamond carbon formation. Compared with our previous results obtained with IR laser vibrational excitation, the UV laser irradiation acted in a nonthermal fashion, in which reactive species for diamond growth were directly produced through photolysis. The systematic investigation of how the energy coupling path, either through vibrational or electronic excitation, affects the diamond growth process provides a clear guideline for fully exploring the advantages of laser chemistry in material synthesis.

## Materials and methods

A schematic diagram of the UV-laser-assisted combustion diamond CVD experiment setup is shown in Supplementary Fig. S1. A detailed illustration of the diamond growth process is provided in the Supplementary Information. A combustion flame generated from a mixture of ethylene (C_2_H_4_), acetylene (C_2_H_2_) and oxygen (O_2_) was used for diamond growth. A UV krypton fluoride (KrF) excimer laser beam (Lambda Physik, COMPex 205, Santa Clara, CA, USA) with a wavelength of 248 nm and a pulse width of 23 ns was directed perpendicularly through the combustion flames and parallel to the substrate. Diamond growth was performed using UV laser irradiation of the combustion flame by tuning the laser fluence from 0.6 to 1.4 J cm^−2^ at a laser frequency of 35 Hz. To investigate the growth rate and film quality, the deposition time was varied to obtain a similar film thickness, ~10 μm, for comparison purposes. For the diamond nucleation study, the deposition time was 10 min. The setup for the OES and LIF study was similar to that reported in a previous work^19^. A detailed schematic drawing of the setup and the measurement parameters are provided in Supplementary Fig. S2.

The surface morphologies and cross-sectional microstructures of the diamond films were characterized by a scanning electron microscope (SEM, XL-30, Philips Electronics Optics, Eindhoven, Netherlands). The growth rate was calculated by dividing the film thickness by the deposition time. Diamond quality was evaluated using a micro-Raman spectrometer (inVia, Renishaw, New Mills, UK). An argon-ion laser with a wavelength of 514.5 nm and a power of 50 mW was used as the exciting source. The beam was focused to a spot size of ∼5 μm using a 20 × objective lens. TEM (FEI Tecnai Osiris, 200 kV, Thermo Fisher Scientific Inc., OR, USA) was performed to study the growth transition zone at the film–substrate interface.

## Results and discussion

Material synthesis often proceeds through chemical processes occurring in the gas phase^20^. Examination of the variation in the gas phase under UV laser irradiation is therefore critical in determining the role of irradiation in the growth process. As one of the most commonly used techniques for detecting excited species in a reaction system, OES of diamond-forming flames under different laser irradiation conditions was studied. Supplementary Fig. S3a illustrates the acquisition time sequences between the KrF UV laser and the intensified charge-coupled device gate of the spectrometer. The emission peaks from three mains species were detected in the spectra (Supplementary Fig. S3b): (1) C_2_: A^3^Π_g_→X’^3^Π_u_ (Δ*v*=−1, 0, 1, 2), (2) CH: A^2^Δ→X^2^Π (Δ*v*=0) and (3) OH: A^2^Σ^+^→X^2^Π (Δ*v*=0)^19^. All species’ emission peaks grew as the laser fluence increased. The OES peak intensities assigned to each excited species were integrated and summed, representing their abundance in the flame.

**Figure 2:**
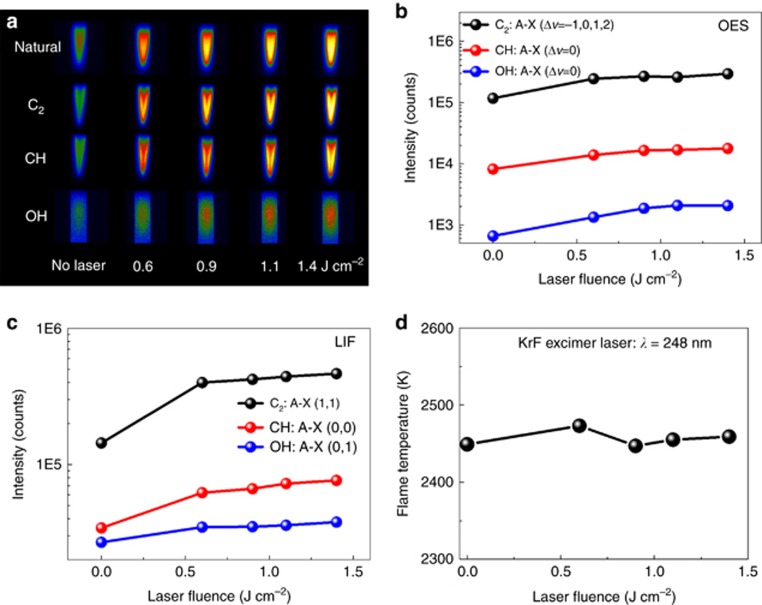
Images, chemistry and temperatures of combustion flames under UV laser irradiations with respect to the laser fluence. (**a**) Flame images without and with UV laser irradiation at different laser fluences with different filters inserted. Integrated intensities of (**b**) OES peaks and (**c**) LIF signals of C_2_, CH and OH plotted as a function of the laser fluence. (**d**) The flame temperature plotted as a function of the laser fluence.

It was difficult to determine two-dimensional distributions of the species using spectroscopic diagnostics with point measurements^21, 22^. Two-dimensional natural emission flame images under different UV irradiation conditions provided more insight into the radical distribution within the flame, as shown in Figure 2a. False coloring was used to indicate the emission intensities, representing the excited species’ abundance. Notably, the total emission from the flame became brighter as the laser fluence increased. Three filters were placed in the optical path to measure the two-dimensional emission images created by the emission band from each excited species: diatomic carbon (C_2_), methylidyne radical (CH) and hydroxyl radical (OH). Upon inspection of the images, it was clear that UV laser irradiation effectively induced generation of all three excited species, following the same trend demonstrated in the spectra. It was noted that the increase in OH was concentrated in the center of the flame, while the amounts of C_2_ and CH were more significant at the flame edges.

A limitation of OES is that it only probes excited-state species, which are usually not important in the overall chemistry of a reaction because their number densities are several orders of magnitude smaller than those of the ground state. LIF is often the technique of choice for selectively and unambiguously detecting ground-state species by their excitation spectrum, and the LIF signal is directly proportional to the density of the probed ground-state species. The detailed LIF measurement procedure and results are shown in the Supplementary Information. The LIF signals of C_2_ (A-X (1,1)) at 512.9 nm, CH A-X (0,0) at 431.4 nm and OH A-X(0,1) at 347.2 nm by exciting C_2_ (X-A (0,1)) at 473.7 nm, CH (X-B (0,0)) at 388.9 nm and OH (X-A (0,0)) at 307.8 nm were separately observed, as shown in Supplementary Fig. S4. The LIF signals assigned to each ground-state species were integrated, representing their abundance in the flame.

The integrated intensities of OES peaks and LIF signals of C_2_, CH and OH are plotted as a function of the laser fluence in Figure 2b and 2c. Compared with the values obtained without UV laser irradiation, significant increases in the integrated peak intensities for both excited- and ground-state species (C_2_, CH, OH) were observed with UV laser irradiation, suggesting that more excited- and ground-state species were generated with UV irradiation. However, the increase in the integrated peak intensities decreased as the laser fluence increased from 0.6 to 1.4 J cm^−2^.

Distinct increments in the concentrations of both excited- and ground-state C_2_, CH and OH species were observed in the UV-laser-irradiated flame. These species are all considered to be critical for diamond formation^23, 24, 25, 26, 27^. The next pertinent question that arises is, ‘How were these species generated? By pyrolysis or photolysis?’ To determine how these species were generated, the flame temperature, *T*_flame_, was analyzed at different laser fluences. Due to strong coupling between the translational and rotational energy states, the flame temperature can be approximated by the rotational temperature derived from a high-resolution rotational line emission intensity of CH using the Boltzmann plot^28, 29^. The detailed calculation procedure is illustrated in Supplementary Fig. S5. As shown in Figure 2d, the flame temperature was found to remain nearly constant with respect to the laser fluence, suggesting that the UV-laser-induced generation of both excited- and ground-state species (C_2_, CH and OH) was attributed to nonthermal processes: electronic excitation and direct photolytic processes. This finding is significantly different from what we observed with IR laser vibrational excitation, in which the flame temperature increased by 252 K under wavelength-matched laser irradiation, as shown in Figure 1 and Ref. 7.

**Figure 3:**
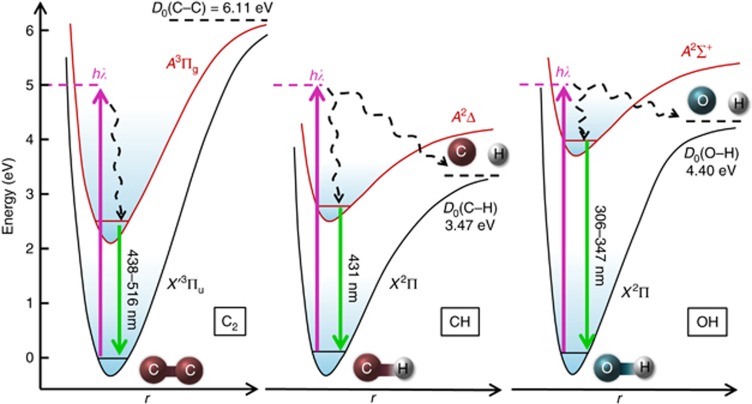
Diagram of transition processes of reactive radicals, C_2_, CH and OH, under UV laser excitation.

The difference, in terms of flame temperature variation, suggests completely distinct energy coupling mechanisms of IR laser and UV laser photochemistry. UV photochemistry is a ‘cold’ process, contributing little to the gas-phase temperature variation. As illustrated in Figure 3, UV photons absorbed by the molecules can directly excite electronic transition to the state in which dissociation is ready to occur when the photon energy, *hv*, exceeds the bond dissociation energy, *D*_0_ (Ref. 3). The photon energy of the UV laser, 5 eV, was high enough to induce both electronic excitations of CH, OH and C_2_ and direct photolysis of CH and OH, whose bond dissociation energies were lower than the UV photon energy (Figure 3). Based on the comparison of LIF and OES signals, it was found that both electronic excitation and photolysis occurred in the flame with UV laser irradiation.

In addition to the three emissive species that were detectable using OES and LIF, there was a large number of other nonemissive hydrocarbons existing in the flame, including CH_3_ (*D*_0_=4.69 eV) and C_2_H_4_ (*D*_0_=4.81 eV), whose bonding energies were lower than the UV photon energy^30^. Photolysis of C_2_H_2_ and more than 20 organic molecules by either a 193- or 248-nm laser has also been reported previously^14, 15^. The higher the laser fluence is, the greater the photon flux becomes, leading to the generation of more reactive species. This situation is different from what occurred with IR laser irradiation in our previous work. IR laser energy was coupled into the gas phase through vibrationally exciting precursor molecules. The fact that IR photon energy, 0.12 eV, is much lower than the bond-breaking energy of most hydrocarbon species, 3–6 eV, suggests that direct molecular dissociation by simply absorbing IR photons is less likely. As shown in Figure 1, IR-laser-induced excitation promotes targeting molecules from the vibrational ground state to the excited state but within the same ground electronic manifold. Rapid intermolecular and intramolecular vibrational energy transfer serves as a heat bath for enhancing the reactivity of precursor molecules and accelerating chemical reactions. Released heat from the accelerated reactions contributes to an increase in flame temperature with IR laser irradiation^3^. Therefore, IR laser chemistry should be viewed as a ‘hot’ process compared with ‘cold’ UV laser photochemistry, as indicated in Figure 1. Instead of pooling energy to drive chemical reactions and subsequently producing critical species for diamond formation, the electronic excited reactive species and active species were generated from direct UV photolysis in a ‘nonthermal’ fashion.

**Figure 4:**
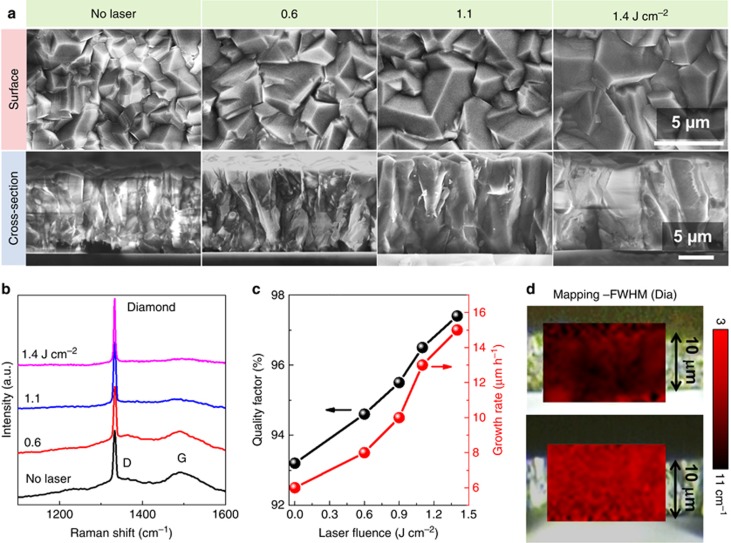
Microstructure, growth rates and quality factors of diamond films prepared with laser irradiations with respect to the laser fluence. (**a**) SEM images of surface and cross-sectional morphologies of diamond films deposited without and with UV laser irradiation at different laser fluences and 35 Hz. (**b**) Raman spectra of corresponding diamond films. (**c**) The growth rate and the film quality factor plotted as a function of the laser fluence. (**d**) Raman mapping in terms of diamond peak full-width at half-maximum of the diamond films prepared (top) without and (bottom) with laser irradiation at 1.4 J cm^−2^.

Based on an understanding of the variation of the flame gas phase with UV laser irradiation, we prepared diamond films under UV laser irradiation at a fixed laser frequency of 35 Hz with the laser fluence tuned from 0.6 to 1.4 J cm^−2^ to study the influence of UV laser irradiation on diamond growth. The surface morphologies of the diamond films deposited at different laser fluences, from 0.6 to 1.4 J cm^−2^, are shown in Figure 4a. The diamond film deposited without UV laser irradiation consisted of randomly oriented grains with an average size of 2 μm. The average diamond grain size increased with the laser fluence, reaching a value of 5 μm at a laser fluence of 1.4 J cm^−2^.

CVD diamond grows as a columnar structure with a grain size that is initially very small and that increases through the film thickness^31^. To determine how UV laser irradiation affects the grain size evolution, the cross-sectional microstructure of the films was evaluated. As shown in Figure 4a, large, uniform grains were obtained with UV laser irradiation, and the lateral grain size increased with the laser fluence. The fast lateral grain size evolution suggests that diamond crystal growth under UV laser irradiation encountered less secondary nucleation, which is known to impede expanded growth of a single grain. Secondary nucleation is believed to arise from amorphous graphitic carbon accumulation that alters the initial crystal growth direction and subsequently induces secondary nucleation^32^. The significantly enlarged lateral grain size indicated that UV laser irradiation greatly suppressed the formation of amorphous graphitic carbon during diamond growth.

Diamond film quality was evaluated using Raman spectroscopy. A sharp diamond peak at ∼1332 cm^−1^ was observed in all spectra, as shown in Figure 4b. The D-band at 1370 cm^−1^ and the G-band centered at 1500 cm^−1^ represent the disordered carbon content and graphitic carbon content in the films, respectively^33^. The diamond peak became sharper and more intense as the laser fluence increased, suggesting that higher diamond quality and a purer diamond phase were obtained with UV laser irradiation. A quality factor, *Q*_i_=*I*_diamond_/(*I*_diamond_+*I*_a-carbon_/233), was derived from the Raman spectra, where *I*_diamond_ and *I*_a-carbon_ are the integrated intensities of the diamond peak and the sum of the integrated intensities of the nondiamond carbon bands, respectively^34^. The diamond quality factor exhibited a nearly linear increase with respect to the laser fluence, reaching a value of 97.4% at a laser fluence of 1.4 J cm^−2^, 4.2% higher than that of the film prepared without laser irradiation (Figure 4c). The large diamond grains observed in the films prepared by UV laser irradiation at a high fluence led to the improved diamond quality because of a weaker contribution from the grain boundaries where nondiamond carbon formed. The narrowing diamond Raman peak was also closely related to the large diamond grains obtained with UV laser irradiation, through which the detecting laser light encountered less deformation. The growth rate, *R*, showed a linear increase with respect to the laser fluence, as shown in Figure 4c, reaching a value of 15 μm h^−1^ at 1.4 J cm^−2^, more than twice the value obtained without UV laser irradiation. The enhanced growth rate and the improved quality suggest the positive role of UV laser irradiation in promoting diamond growth.

Raman mapping based on the full-width at half-maximum of the diamond Raman peak was performed to evaluate the cross-sectional grain crystal quality and uniformity. As shown in Figure 4d, the cross-section of the diamond film prepared with UV laser irradiation at 1.4 J cm^−2^ was more uniform than that prepared without laser irradiation, exhibiting an average full-width at half-maximum value of 5.7 cm^−1^, 3.1 cm^−1^ narrower than that prepared without a laser. A uniform increase in diamond quality was thus confirmed through the film thickness with UV laser irradiation.

**Figure 5:**
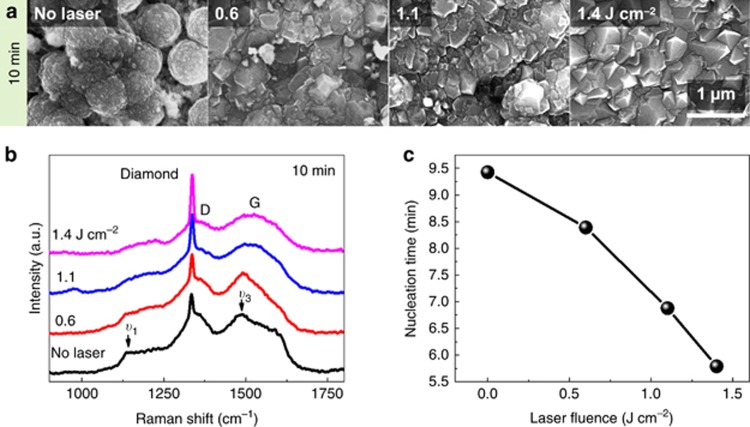
Morphology and diamond quality of diamond films prepared at the nucleation stage under UV laser irradiations and the total nucleation time with respect to the laser fluence. (**a**) SEM images of diamond film surface morphologies prepared without and with UV laser irradiation at different laser fluences and 35 Hz for 10 min. (**b**) Raman spectra of the corresponding diamond films. (**c**) The nucleation time plotted as a function of the laser fluence.

The morphology and quality characterizations of the diamond films suggested that UV laser irradiation affected the diamond growth process such that it suppressed graphitic carbon formation and favored diamond growth. The nucleation stage is the most sensitive to amorphous graphitic carbon accumulation because diamonds nucleate from spontaneous precipitation of a pure *sp*^*3*^ carbon cluster from the amorphous graphitic carbon matrix that forms during the incubation stage^35, 36^. To confirm the suppressing effects of UV laser irradiation on amorphous graphitic carbon accumulation, the diamond nucleation process was further investigated by *in situ* monitoring of the field-enhanced thermionic emission current^36^ and preparing diamond films within a short period, that is, 10 min. The current measurement setup and the thermionic current evolution as a function of the deposition time are illustrated in the Supplementary Information. The surface morphologies of the 10-min diamond films prepared at different laser fluences are shown in Figure 5a. Cauliflower-like nanodiamond films were obtained without laser irradiation, while UV laser irradiation transformed the nanodiamond features into larger, faceted microcrystal structures. The cauliflower-like grains formed under conditions in which continuous growth of initially formed crystalline nuclei was severely disturbed by the secondary nucleation process due to the accumulation of amorphous graphitic carbon. As indicated by the Raman spectra (Figure 5b), the 10-min diamond film prepared without laser irradiation exhibited a typical nanodiamond feature. The Raman peaks appearing at ∼1150 (*υ*_1_) and 1480 cm^−1^ (*υ*_3_) are related to the trans-polyacetylene phase present at the grain boundaries as the grain size reached the nanometer scale, representing a signature of nanodiamond^33^. These two peaks became weaker, and the diamond peak rose as the laser fluence increased. At a laser fluence of 1.4 J cm^−2^, the *υ*_1_ and *υ*_3_ peaks were difficult to retrieve, and a microdiamond Raman feature was exhibited.

As shown in Figure 5c, the nucleation period was clearly shortened from 9.5 to 5.5 min as the laser fluence increased. Diamond nuclei grow through transformation of amorphous carbon into diamond at the amorphous matrix–diamond interface. This transformation is governed by a kinetic balance between the etching rate and the growth rate of graphitic carbon and diamond^36^. The longer the nucleation process is, the thicker the amorphous carbon transition layer that forms at the initial stage becomes. The significantly shortened nucleation process confirmed that the UV laser irradiation affected the diamond growth process by quickly balancing the kinetic competition process such that it favored diamond nucleation and suppressed amorphous graphitic carbon codeposition. The significantly reduced nucleation time and the morphology/Raman characterization of the 10-min diamond films suggest that UV laser irradiation helped complete the nucleation process quickly, which was accompanied by greatly suppressed nondiamond carbon accumulation.

**Figure 6:**
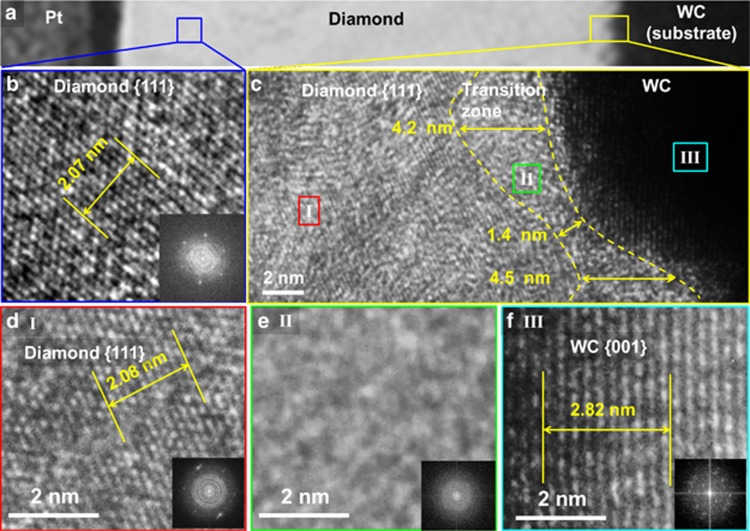
TEM microstructure characterization of the film-substrate interface of a diamond film prepared with UV laser irradiation. (**a**) TEM image of a 389-nm thick diamond film on a WC substrate prepared with UV laser irradiation at 1.4 J cm^−2^. HRTEM images of (**b**) the diamond crystal region with its FFT pattern and (**c**) the film–substrate interface, as indicated by a blue square and a yellow rectangle in **a**, respectively. Atomic-resolution TEM images of the regions (**d**) I, (**e**) II and (**f**) III marked in **c** with their corresponding FFT patterns, representing diamond, the transition zone and the substrate, respectively.

Figure 6a shows a TEM image of the cross-section of a 389-nm thick diamond film on WC prepared with UV laser irradiation at 1.4 J cm^−2^. A thin platinum (Pt) layer was deposited on the diamond film to protect it from ion beam damage during sample preparation. A high-resolution TEM (HRTEM) image (Figure 6b) of the diamond crystal region marked by a blue square, in Figure 6a, clearly shows continuous diamond {111} family planes, confirming the good diamond grain quality. Diamond has an interplane *d*_111_ value of 2.06 Å. A distance of 10 planes was measured to accurately determine the *d* value of diamond {111} planes, 2.08 nm, well matching the theoretical value. The corresponding fast Fourier transform (FFT) pattern shows diamond (440) spots, suggesting the beam direction was [110].

**Figure 7:**
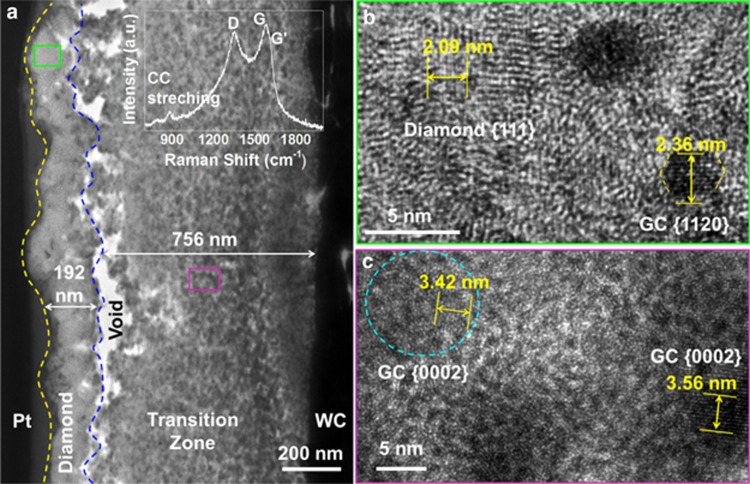
TEM microstructure characterization of the film-substrate interface of a diamond film prepared without UV laser irradiation. (**a**) TEM image of a diamond film prepared without UV laser irradiation and HRTEM images of (**b**) the diamond crystal region and (**c**) the nondiamond carbon transition zone marked by green and purple rectangles in **a**, respectively. The inset in **a** is a Raman spectrum collected from a cross-section of the TEM sample.

To further confirm that the nucleation time was greatly reduced and the amorphous carbon transition layer was suppressed with UV laser irradiation, HRTEM was performed to reveal the film–substrate interface of the diamond film prepared with UV laser irradiation in Figure 6c. No sharp interface was identified because the tungsten carbide (WC) substrate was not atomically flat and had nanometer-scale roughness. Tungsten carbide has a theoretical *d*_001_ value of 2.82 Å. Based on the atomic-resolution HRTEM images (Figure 6d–6f) of different zones and their FFT patterns, shown in the inset, it is easy to differentiate the zones based on the calculated *d* value. As shown in Figure 6c, the average transition zone thickness of the diamond film prepared with UV laser irradiation was ∼4 nm, which was significantly narrower than that observed for the 765-nm thick graphite carbon zone between the diamond coating and the WC substrate in the diamond film prepared without UV laser irradiation, as shown in Figure 7a. The TEM diamond sample prepared without UV laser irradiation exhibited a typical polycrystalline graphitic carbon feature, as indicated in the Raman spectrum (the inset of Figure 7a). An HRTEM image (Figure 7b) of the diamond crystal region marked by a green rectangle in Figure 7a shows fringes from the diamond {111} planes with some particle inclusions, which can be assigned to graphitic carbon quantum dots with {1120} planes according to the measured *d* value. An HRTEM image of the transition zone marked by a purple rectangle in Figure 7a shows graphitic carbon particles embedded in an amorphous matrix, matching the Raman spectroscopy results. This narrow transition zone in the diamond film prepared with UV laser irradiation was comparable to that in the diamond films on WC prepared using microwave-enhanced CVD and hot-filament CVD, as previously reported^37, 38^, which are known to produce higher-quality diamond films than combustion CVD. The narrow transition zone observed with UV laser irradiation again confirms that UV laser irradiation altered the diamond growth process by suppressing the formation of nondiamond carbon.

The observation of fast lateral grain size evolution and the significantly reduced nucleation time can be explained by the observation of gas-phase variation in the flame. Figure 2a shows a pronounced increase in OH abundance in the central region of the flame. OH radical plays a critical role in combustion synthesis of diamond by etching surface-bound hydrogen and stabilizing *sp*^*3*^-hybridized surface carbon bonds^23, 24^. The increase in the amount of OH radical explains why graphitic carbon accumulation was greatly suppressed by UV laser irradiation. Another possible contribution to the significantly promoted diamond growth is an increased atomic H concentration due to promoted H-abstraction processes with UV laser photolysis. The photoinduced dissociation of hydrocarbons preferentially occurs at C–H bonds through H-abstraction processes rather than at C–C bonds^7^. The primary processes of 193 nm UV photolysis of C_2_H_2_ are^39^


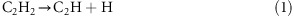



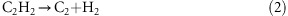


The primary processes of 147 nm UV photolysis of C_2_H_4_ are^40^


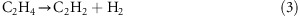



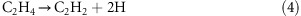


The reactions of atomic hydrogen not only control the gas-phase chemistry but also determine the availability of reactive sites. Diamond growth from an oxyacetylene flame proceeds through two critical surface reactions: (1) addition of reactive hydrocarbon radicals to the active surface sites and (2) H-abstraction from hydrocarbon radicals to create more reactive sites to accept hydrocarbons and stable *sp*^*3*^-hybridized carbon bonds^31^. The H-abstraction processes induced by UV laser photolysis of hydrocarbons thus could play a dominant role in promoting diamond growth. However, a more detailed study is required to confirm this hypothesis because the KrF excimer laser used in this study has a longer wavelength, 248 nm, than that of lasers used in the reported photochemistry study of C_2_H_2_ and C_2_H_4_, making the photoinduced H-abstraction rate much smaller^39, 40^.

Compared with the diamond growth results obtained with two different energy coupling paths, IR vibrational excitation and UV photolysis in Figure 1, IR vibrational excitation acted thermally to enhance the reactivity of reactant molecules and accelerate the total reaction rate, while UV photolysis contributed nonthermally to the diamond growth process by providing critical reactive species through direct molecular photofragmentation. Both energy coupling paths promoted the diamond growth rate and quality simultaneously. However, compared with the continuous IR laser energy input using a continuous-wave CO_2_ laser, the interaction time between the UV laser and the diamond-forming flame was determined by the laser pulse width, 23 ns, but with an extremely high peak power density of 61 MW cm^−2^. Under such high peak power, multiphoton absorption leading to molecular photofragmentation is achievable. The lifetime of the photon-induced reactive species, C_2_ and CH, were measured using time-resolved OES with an intensified charge-coupled device gate width of 200 ns. The detailed measurement procedure is illustrated in the Supplementary Information and Supplementary Fig. S7. The deduced lifetimes of C_2_ and CH were 0.7 and 1.6 μs, respectively. Taking the laser repetition rate of 35 Hz into consideration, the actual UV impact time occupied only 5.7 × 10^−2^% of the total growth time. Although it is still not clear why, it is impressive that such a short-lived effect of the gas significantly altered diamond growth on a much larger time scale. Compared with UV laser irradiation, IR laser irradiation produced a more pronounced increase in the diamond growth rate and diamond quality, which could be attributed to the enormously different power capabilities of the lasers. The UV KrF excimer laser used for the photolysis study outputs up to 7 W of power, two orders of magnitude lower than the power output of the IR CO_2_ laser used for the vibrational excitation study, 1800 W. However, the fact that the growth rate increase under UV laser irradiation was comparable to that under IR laser irradiation suggests that the ‘cold’ electronic energy coupling achieved using UV laser irradiation affects the combustion process more efficiently than the ‘hot’ vibrational energy coupling achieved using IR laser irradiation.

## Conclusions

The influence of UV-laser-induced photolysis on diamond growth was investigated. OES and LIF of the flame indicated that UV-laser-induced photolysis produced large amounts of reactive radicals in the flame, contributing directly to the promotion of diamond growth. Investigation of the nucleation process suggested that UV laser irradiation modified diamond growth such that it favored diamond formation and suppressed nondiamond carbon accumulation, leading to an enhanced diamond deposition rate and improved diamond quality. The diamond growth observed under UV laser irradiation was compared with that assisted by IR vibrational excitations. The results suggest that both energy coupling paths facilitated diamond growth to some extent, although the working mechanisms were completely distinct. The discovery of the advantages of laser photochemistry in diamond growth is of great significance for vastly improving the synthesis of a broad range of technically important materials.

## Author contributions

The manuscript was written through contributions of all of the authors. All authors have given approval for the final version of the manuscript.
