# Supplementary material for: Ultraviolet laser photolysis of hydrocarbons for nondiamond carbon suppression in chemical vapor deposition of diamond films
Source: Light Sci Appl. 2018 Apr 6;7:17177–. doi: 10.1038/lsa.2017.177 (PMC6060054; doi:10.1038/lsa.2017.177)
Supplement: Supplementary Information [file lsa2017177x1.docx]

Supplementary Information

Ultraviolet laser photolysis of hydrocarbons for nondiamond carbon suppression in chemical vapor deposition of diamond films

Lisha Fan,^1,^ ^‡^ Loic Constantin,^1,2,^ ^‡^ Dawei Li,^1^ Lei Liu,^1^ Kamran Keramatnejad,^1^ Clio Azina, ^1,2^ Xi Huang,^1^ Hossein Rabiee Golgir,^1^ Yao Lu,^1^ Zahra Ahmadi,^3^ Fei Wang,^3^ Jeffrey Shield,^3^ Bai Cui,^3^ Jean-Francois Silvain,^2^ and Yong Feng Lu^1,^*

^1^Department of Electrical and Computer Engineering, University of Nebraska-Lincoln, Lincoln, NE 68588, USA;

^2^Institut de Chimie de la Matière Condensée de Bordeaux—ICMCB-CNRS 87, Pessac, 33608, France;

^3^Department of Mechanical and Materials Engineering, University of Nebraska-Lincoln, Lincoln, NE 68588, USA

*Correspondance : Prof. Yongfeng Lu ; Address : Electrical and Computer Engineering Department, University of Nebraska-Lincoln, 209N Walter Scott Engineering Center, Lincoln, NE, 68588; Tel: 402-472-8323; Fax: 402-472-4732; Email: ylu2@unl.edu.

1. **UV-laser-assisted diamond combustion chemical vapor deposition**

**Figure S1** shows the schematic diagram of the ultraviolet (UV)-laser-assisted diamond combustion chemical vapor deposition (CVD) setup. A combustion torch with a 1.5 mm orifice tip was used to produce the flames. The precursor gases were a mixture of ethylene (C_2_H_4_, 99.999%), acetylene (C_2_H_2_, 99.6%), and oxygen (O_2_, 99.996%) with a volume ratio of 1:1:2. A UV krypton fluoride (KrF) excimer laser beam (Lambda Physik, COMPex 205), with a wavelength of 248 nm and a pulse width of 23 ns, was directed perpendicularly through the combustion flames and parallel to the substrate to excite the combustion species. A UV convex lens with a focal length of 25 mm was used to focus the laser beam from its original size of ~ 20 × 10 mm^2^ to ~ 2 × 1 mm^2^, which was enough to cover the whole primary flame. A power meter was placed in the UV laser path next to the combustion flame to measure the laser power. The laser fluence was calculated by dividing the laser pulse energy by the beam spot size. Diamond growth was performed with UV laser irradiation of the combustion flame by tuning the laser fluence from 0.6 to 1.4 J/cm^2^ at a laser frequency of 35 Hz. A tungsten carbide (WC) with a cobalt composition of 6% and a dimension of 12.5×12.5×1.6 mm^3^ was placed on a water-cooled brass plate that was mounted on an X-Y-Z stage. The distance between the substrate surface and the primary flame tip was kept around 0.5 mm in all experiments. The substrate temperature during the deposition was maintained at 770 ~ 780 ℃ and monitored by a noncontact pyrometer (OS3752, Omega Engineering, Inc.). For growth rate and film quality investigation, the deposition time was varied to obtain a similar film thickness, ~ 10 μm, for comparison purposes. For the diamond nucleation study, the deposition time was 10 min.


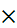

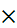


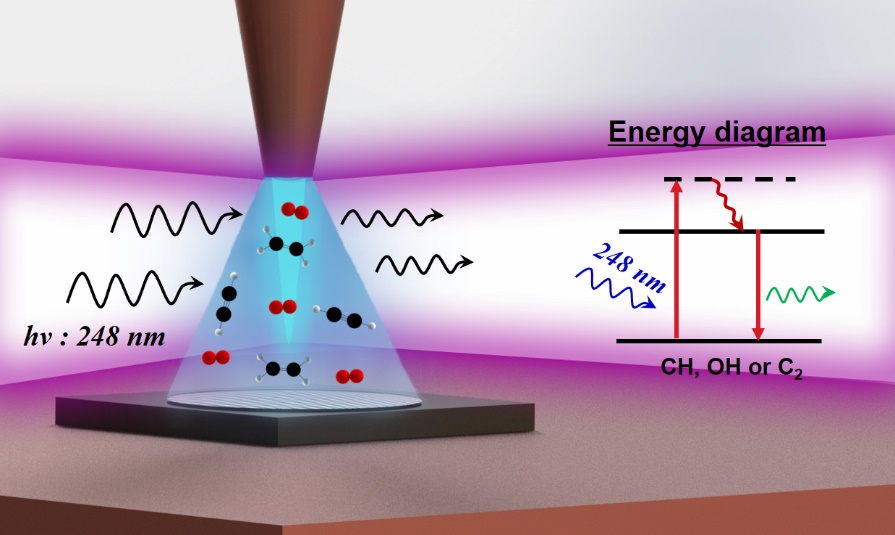


Figure S1. Schematic illustration of the UV-laser-assisted diamond combustion CVD setup.

1. **Gas phase investigation of the combustion flame assisted with UV laser irradiation**

Schematic diagram of the OES and LIF measurement setup to fully characterize flame species at both the excited and ground states, respectively, is shown in **Figure S2**.

For OES measurement, a KrF Excimer laser with a wavelength of 248 nm and a repetition rate of 10 Hz was used for irradiating the flames. The laser fluence was tuned from 0.6 up to 1.4 J/cm^2^. The laser beam was focused to a spot size of 4 mm × 8 mm on the sample surface by Lens 1 (f1 = 20 cm) to fully cover the primary inner flame. Lens 3 (f3 = 10 cm) was used to collect and couple the emission from excited molecules into a spectrometer (Andor Technology, Shamrock 505i, intensified charge-coupled device (ICCD) DT-334T, 3 gratings: 150, 600, 2400 l/mm, range: 190-800 nm) for measurement. The laser and the ICCD of the spectrometer were synchronized using a digital delay generator (Stanford Research System DG 535, 5 ps delay resolution). The following parameters were used for OES spectrum and flame image measurement with KrF UV laser irradiation at different laser fluences: ICCD gate delay = 0 μs, ICCD gate width = 10 μs, and KrF laser frequency = 35 Hz. The wide-range spectra for species identification were collected with a horizontal slit width of 50 μm centered at the primary flame using gratings of 150 lines/mm while the high-resolution narrow-range spectra for rotational temperature calculation were collected using a grating of 2400 lines/mm. The horizontal slit width was set to 2500 μm for taking flame images. The flame images were proportional to the real flames in size. All of the spectra and images were measured for 500 pulses to reduce the standard deviation in this study. A background spectrum was taken before the collections of the emission spectra and subtracted from all captured spectra.


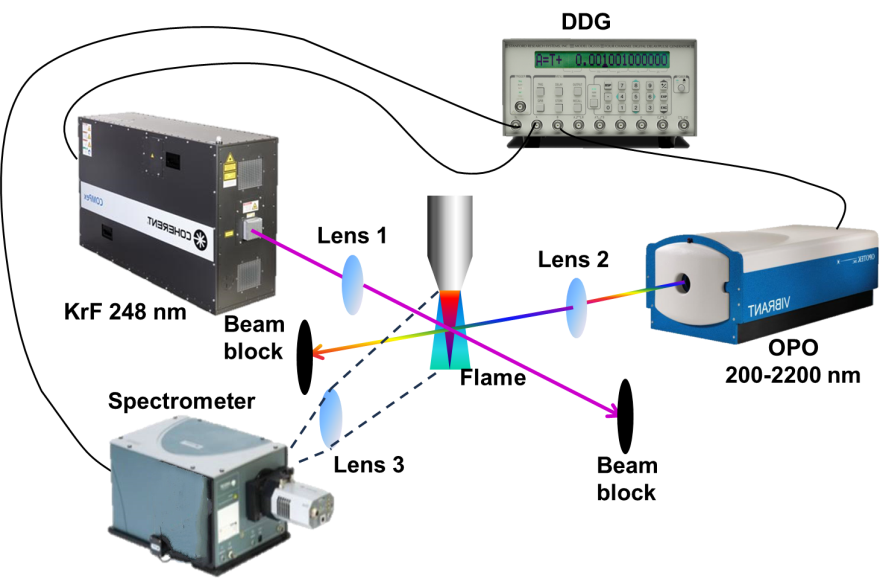


Figure S2. Schematic diagram of the optical emission spectroscopy (OES) and laser-induced fluorescence (LIF) setup to characterize the species in the combustion flame with UV laser irradiation.

For LIF measurement, a wavelength tunable optical parametric oscillator (OPO) laser (OPOTEK Inc., VIBRANT^™^ 355 LD, 5 ns, 0.22–2.4 µm) was slightly defocused on the flame by Lens 2 (f2 = 10 cm) to a spot size of 5 mm in diameter. The probe laser wavelength was tuned to selectively excite the electronic transition system of intermediate species (C_2_, CH, and OH) presenting in the diamond-forming flame. The probe laser energy was 5 mJ/pulse for resonance fluorescence excitation at different wavelengths. The interpulse delay time between the UV KrF laser pulse and the probe OPO laser pulse was controlled by a digital delay generator (Stanford Research Systems DG535, 5 ps delay resolution). The light-collecting lenses were in plane and vertical to the probe laser beam. The ICCD detector was synchronized with the probe laser on time. The following parameters were used for LIF measurement: the interpulse delay time between the KrF UV laser and the OPO laser = 50 ns, ICCD gate delay = 0 ns, ICCD gate width = 5 ns, and laser frequency = 10 Hz. The LIF spectra were collected with a horizontal slit width of 50 μm centered at the primary flame using gratings of 600 lines/mm. All of the spectra were measured for 500 pulses to reduce the standard deviation in this study. Background subtraction is applied to all measurements to eliminate the remaining natural emission of the flame and possible nonresonant fluorescence.

**Figure S3a** illustrates the acquisition time sequences between the KrF UV laser and the ICCD gate of the spectrometer. The emission peaks from three mains species were detected in the spectra: 1) C_2_: X’^3^Π_u_ ← A^3^Π_g_ (Δ*v* = -1, 0, 1, 2), 2) CH: A^2^Δ ← X^2^Π (Δ*v* = 0), and 3) OH: A^2^Σ^+^ ← X^2^Π (Δ*v* = 0) in **Figure S3b**. All species peaks grew as the laser fluence increased. The peak intensities assigned to each excited species were integrated and summed, representing their abundance in the flame.


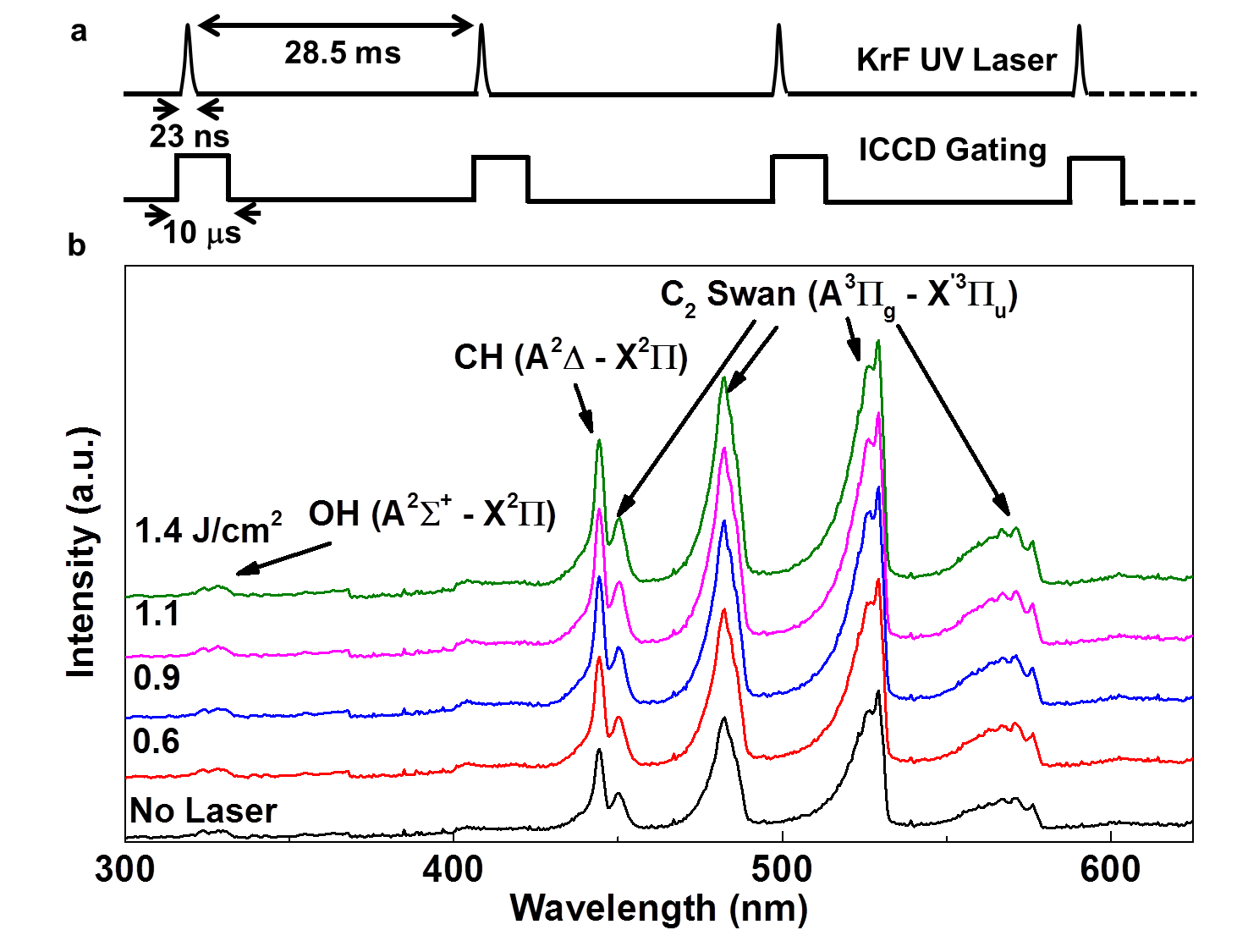


Figure S3. (a) The time relationship between the synchronized ICCD gate of the spectrometer and the KrF UV laser for OES measurement. (b) Optical emission spectra of the combustion flames without and with UV laser irradiation at different fluences at 35 Hz.

The acquisition time sequences among the KrF UV laser, the OPO laser, and the ICCD gating of the spectrometer for LIF measurement are illustrated in **Figure S4a**. **Figures S4b-d** show the electronic transition diagrams, OES spectra, and LIF spectra of C_2_, CH, and OH.

The OES spectra in **Figure S4b** show that the emission intensity in the C_2_ swan band, A^3^Π_g_ - X^’3^Π (Δ*v* = 0), was the strongest at 516.5 nm (A-X (0,0)). The electronic transition diagram in **Figure S4b** shows the exciting of C_2_ radicals through the X^’3^Π (*v*”=0) -> A^3^Π_g_ (*v*’=1) transition by the OPO laser at a wavelength of 473.7 nm. When the excited C_2_ radicals relaxed back to the low state through the A^3^Π_g_ (*v*’=1) -> X^’3^Π (*v*”=1) transition, detection of photons was allowed with a wavelength of 512.9 nm, as indicated in **Figure S4b**. Significant enhancement of the peak intensity at 512.9 nm was observed in the LIF spectra, confirming the effective resonant excitations of the ground state C_2_ species by OPO laser probing.

As shown in **Figure S4c**, CH was excited through the X^2^Π (*v*”=0) -> B^2^Σ^-^ (*v*’=0) transition at 388.9 nm. Because the B^2^Σ^-^ state and the A^2^Δ state crossed each other, strong collisional redistribution between these two states took place. The fluorescence from the A^2^Δ (*v*’=0) -> X^2^Π (*v*”=0) transition was observed around 431.4 nm. Significant enhancement of the peak intensity at 431.4 nm was observed in the LIF spectra, confirming the effective excitations of the ground state CH species by OPO laser probing.

As shown in **Figure S4d**, LIF detection of OH was carried out by excitation of the X^2^Π (*v*”=0) -> A^2^Σ^+^ (*v*’=0) transition at 307.8 nm. Detection of fluorescence around 343-348 nm (A^2^Σ^+^ (*v*’=0) -> X^2^Π (*v*”=1)) was observed.

Quantitative analysis of the LIF signals was impeded by quenching of the fluorescence due to inelastic collision of the excited molecules. Certainly, collisional quenching cannot be neglected in flames used at atmospheric pressure. This quenching depends strongly on the pressure, temperature, and collision partners, which are different in different regions of the flame. The LIF results were, therefore, interpreted in a more qualitative way. The LIF signals at 512.9, 431.4, and 347.2 nm in **Figures S4b-d** were integrated to represent the abundance of the ground state C_2_, CH, and OH species in the flame, respectively.


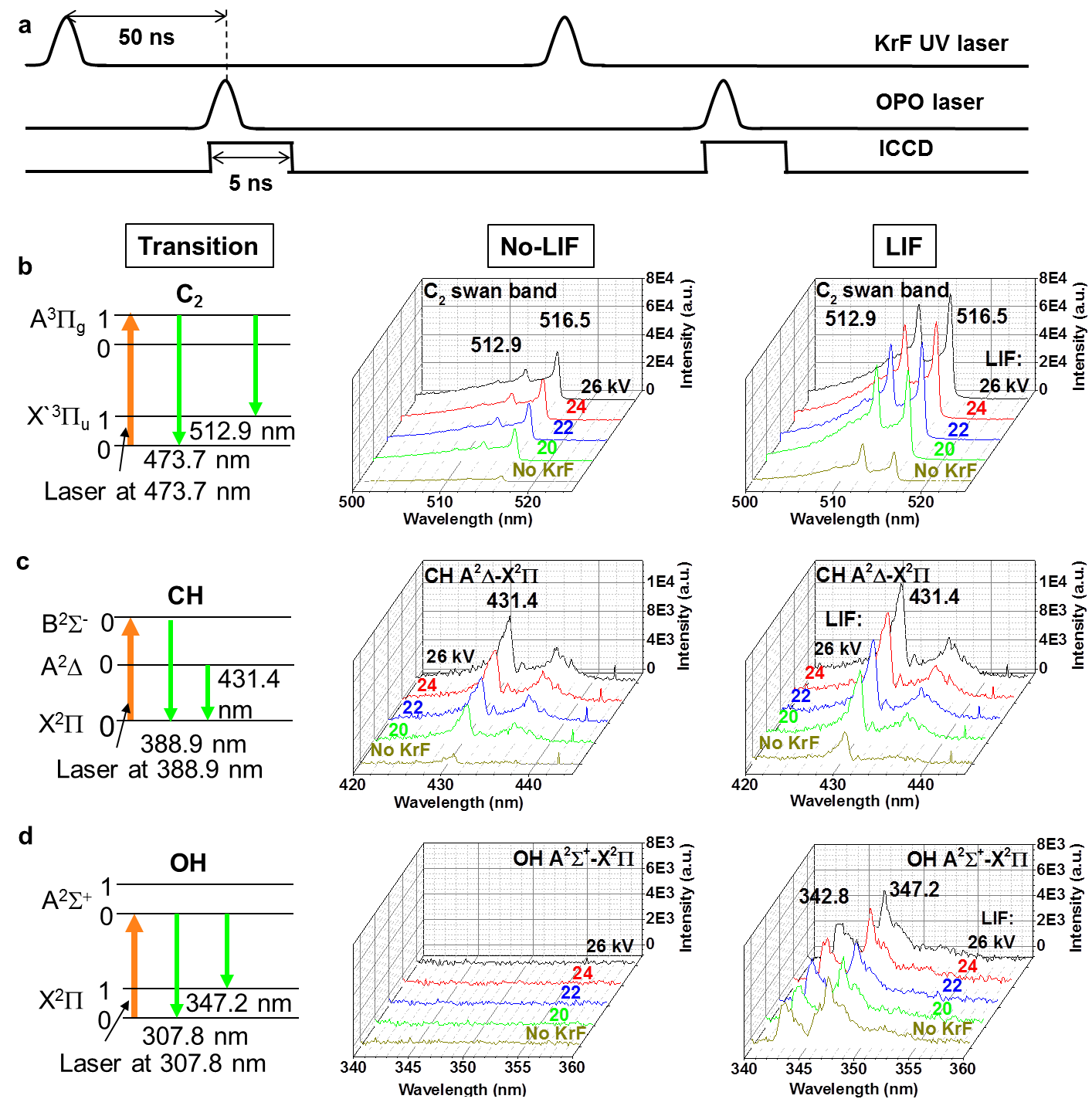


Figure S4. (a) The time relationship among the synchronized ICCD gating of the spectrometer, the KrF UV laser, and the OPO laser for LIF measurement. Electronic transition diagrams, OES and LIF signals of (b) C_2_, (c) CH, and (d) OH with and without UV laser irradiation at different UV laser fluences.

The flame temperature was estimated through the rotational R-branch structure of the CH emission band in the high-resolution optical emission spectra (**Figure S5a**). The Boltzmann plots were derived from the spectra using the following equation:

$$\ln\left( \frac{I\lambda^{4}}{S_{J^{'}J^{''}}} \right)= -\frac{1}{T_{f}}\frac{E_{J^{'}}}{k}+lnC$$

where *I* is the relative emission intensity of a rotational line obtained from the experimental spectrum, *C* is a proportionality constant that is the same for all rotational transitions within a band, *S_J’J”_* is the rotational intensity factor, *λ* is the wavelength of the emitted spectral line, *E_J’_* is the rotational energy of the initial level, *k* is the Boltzmann constant, and *T_r_* is the rotational temperature. **Figure S5b** shows the Boltzmann plots of ln(*Iλ^4^/S_J’J’’_*) versus *E_j’_/k_B_*, in which the slopes correspond to 1/*T_r_*.


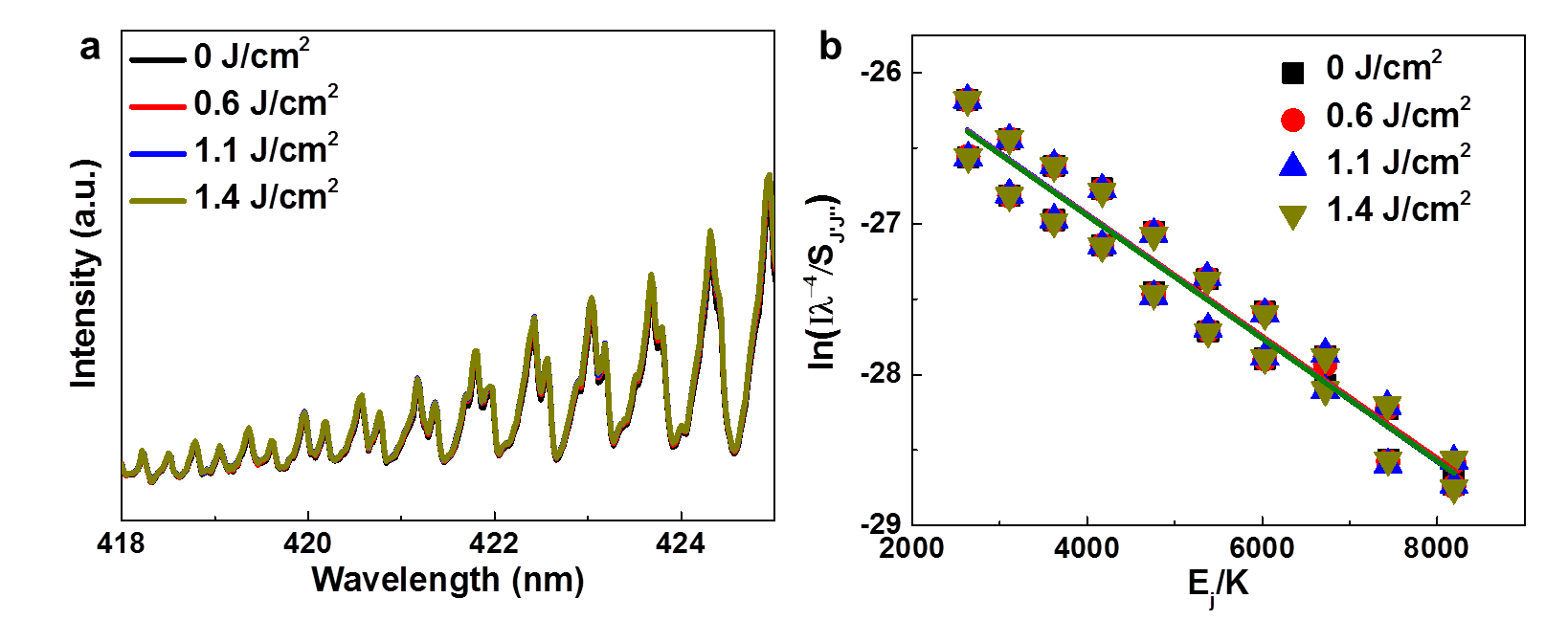


Figure S5. (a) High-resolution optical emission spectra of the rotational R-branch structure of the CH emission band at different laser fluences; (b) Boltzmann plots derived from the optical emission spectra.

1. **Study of diamond nucleation process using thermionic emission current measurement**

**Figure S6a** shows the field-enhanced thermionic emission current measurement setup. A power supply was connected to the flame torch and the brass sample holder. The bias voltage on the substrates was -5 V with respect to the ﬂame torch. The current between the WC substrate and the torch was measured using a nanoampere meter during the diamond growth.


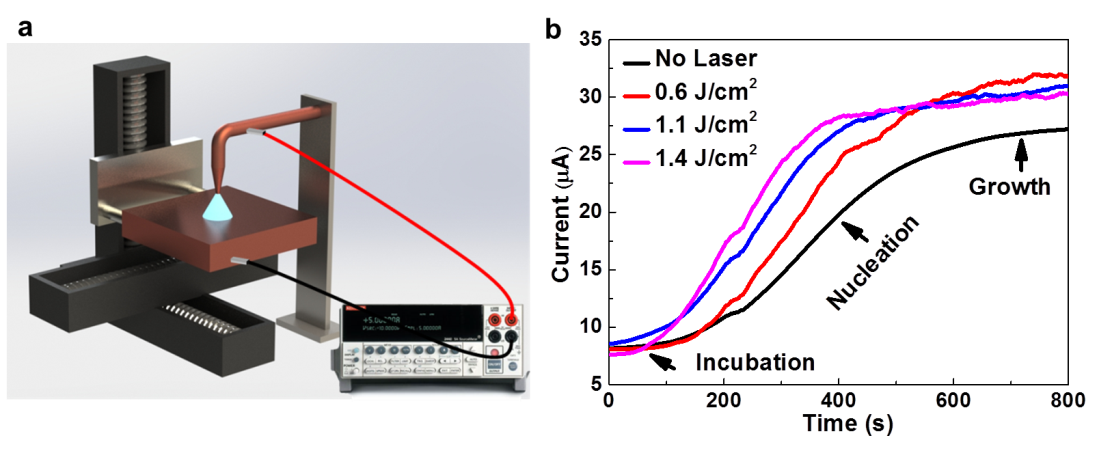


Figure S6. (a) Schematic illustration of *in situ* field-enhanced thermionic current measurement setup. (b) Thermionic current-deposition time curves without and with UV laser irradiation at different fluences.

**Figure S6b** shows the thermionic current-deposition time curves without and with UV laser irradiation at different fluences. The zero-emission current period between 0 to 300 s (the ﬂat segment of the curve) indicates a diamond-free surface, representing an incubation period. From 300 to 600 s, the thermionic current increased from 13 to 27 μA due to newly nucleated diamond particles and increased surface coverage, representing a nucleation period. At 600 s, the thermionic current was close to the maximum, about 28 μA. From 600 to 800 s, the thermionic current remained stable and continuous films form. As shown in **Figure S6b**, the nucleation time was significantly shortened as the UV laser fluence increased.

1. **Measurement of the lifetime of a reactive species**

Time-resolved OES was performed to measure the lifetime of CH and C_2_ under UV radiation at 35 Hz and 1.4 J/cm^2^. The peak intensity of OH was too small to retrieve the lifetime. OES was taken with a gate width of 200 ns and different gate delay times of 0, 0.2, 0.4, 1, 2, and 10 μs, shown in **Figure S7a**. After 10 μs, the spectrum was similar to that without UV radiation, suggesting that each laser pulse affected the flame up to 10 μs. To extract the lifetime of the reactive species, each peak of C_2_ and CH was integrated and summed. **Figure S7b** shows the integrated intensities of C_2_ and CH as a function of the time. The experimental values were fitted exponentially by the equation:


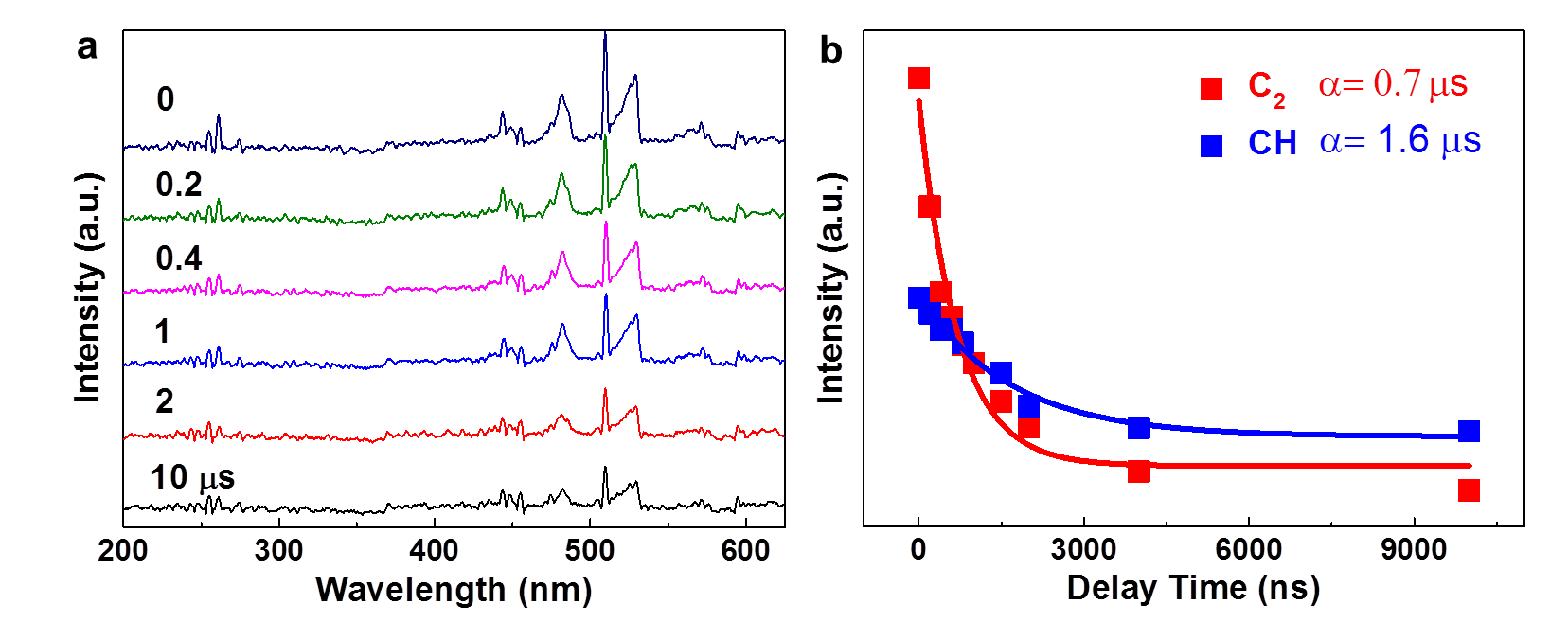


Figure S7. (a) OES with a UV laser irradiation at a different gate delay time: 0–10 μs, gate width was 200 ns; (b) the integrated peak intensities of C_2_ and CH; OES peaks plotted as a function of the delay time.

$$I= e^{-\frac{t}{\alpha}}$$

where *I* is the intensity of each peak, *t* is the delay time, and *α* the lifetime. The measured lifetime of C_2_ and CH were, respectively, 0.7 μs, and 1.6 μs.
